# Association of Preterm Birth With Prescription of Psychotropic Drugs in Adolescence and Young Adulthood

**DOI:** 10.1001/jamanetworkopen.2021.1420

**Published:** 2021-03-12

**Authors:** Christine Strand Bachmann, Kari Risnes, Johan Håkon Bjørngaard, Jorun Schei, Kristine Pape

**Affiliations:** 1 Department of Public Health and Nursing, Faculty of Medicine and Health Sciences, Norwegian University of Science and Technology, Trondheim, Norway; 2 Children’s Clinic, St. Olavs Hospital, Trondheim, Norway; 3 Department of Clinical and Molecular Medicine, Faculty of Medicine and Health Sciences, Norwegian University of Science and Technology, Trondheim, Norway; 4 Department of Research and Development, St. Olavs Hospital, Trondheim, Norway; 5 Faculty of Nursing and Health Sciences, Nord University, Levanger, Norway; 6 Department of Mental Health, Faculty of Medicine and Health Sciences, Norwegian University of Science and Technology, Trondheim, Norway; 7 Department of Child and Adolescent Psychiatry, St. Olavs Hospital, Trondheim, Norway.

## Abstract

**Importance:**

Individuals born preterm have increased risk of mental health impairment compared with individuals born at term. The associations between preterm birth and attention-deficit/hyperactivity disorder and autism are well established; for depression, anxiety, psychotic and bipolar disorder, studies show divergent results.

**Objective:**

To compare the prescription of psychotropic drugs in adolescence and young adulthood between those born preterm and those born at term.

**Design, Setting, and Participants:**

This cohort study used registry data to identify all Norwegians born after 23 weeks of completed gestation between 1989 and 1998. Included individuals were those without registered birth defects, alive at age 10 years, and with available maternal data. Individuals were followed up from 2004 to 2016. Psychotropic drug prescriptions received from age 10 to 23 years were compared between preterm groups and peers born at term. Individuals were compared with their siblings to control for shared family confounding. Data analyses were performed from August 2018 through February 2020.

**Exposures:**

Gestational age at birth **(**GA) was categorized in 4 groups: extremely preterm (GA, 23 weeks and 0 days to 27 weeks and 6 days), very preterm (GA, 28 weeks and 0 days to 31 weeks and 6 days), moderately or late preterm (GA, 32 weeks and 0 days to 36 weeks and 6 days), and full term (GA, 37 weeks and 0 days to 44 weeks and 6 days).

**Main Outcomes and Measures:**

Prescriptions of psychotropic drugs (ie, prescriptions specifically of psychostimulants, antidepressants, anxiolytics, hypnotics or sedatives, or antipsychotics or prescriptions of any of these 5 drugs) among preterm groups were compared with prescriptions among peers born at term and among siblings.

**Results:**

Among 505 030 individuals (259 545 [51.4%] males; mean [SD] birth weight, 3533 [580] g), 762 individuals (0.2%) were extremely preterm, 2907 individuals (0.6%) were very preterm, 25 988 individuals (5.1%) were moderately or late preterm, and 475 373 individuals (94.1%) were full term. Individuals born preterm had increased risk of psychotropic drug prescription, with a dose-response association between GA and prescription. The extremely preterm group had higher rates of prescription for all drug types compared with peers born at term, with odds ratios from 1.7 (95% CI, 1.4-2.1) for antidepressants to 2.7 (95% CI, 2.1-3.4) for psychostimulants. The elevated odds of prescription of all types were less pronounced in the moderately to late preterm group, including odds ratios of 1.1 (95% CI, 1.0-1.1) for antidepressants and 1.2 (95% CI, 1.1-1.2) for psychostimulants. The increases in odds were smaller in the sibling comparison, and increases were not significant for several groups. For example, the OR for any prescription in the sibling analysis was 1.8 (95% CI, 1.2-2.8) in the very preterm group and 1.0 (95% CI, 0.9-1.1) in the moderately or late preterm group.

**Conclusions and Relevance:**

This cohort study found higher rates of prescription of psychotropic drugs throughout adolescence and young adulthood among individuals with all degrees of preterm birth compared with those born at term. These results provide further evidence for an increased risk of mental health impairment among individuals born preterm and suggest that this is not restricted to the most preterm groups.

## Key Points

**Question:**

Do adolescents and young adults who were born preterm receive more prescriptions of psychotropic drugs compared with those who were born full term?

**Findings:**

In this cohort study in Norway of 505 030 individuals followed up from ages 10 to 23 years, all degrees of preterm birth were associated with higher rates of prescription of psychotropic drugs during adolescence and young adulthood compared with those born at term.

**Meaning:**

These findings provide further evidence for increased risk of mental health impairment among individuals born preterm.

## Teaser

This cohort study examines the association between preterm birth and psychotropic drug prescriptions during adolescence and young adulthood.

## Introduction

Children born preterm have an increased risk of neurodevelopmental and cognitive impairment^1,2,3^ and mental and social problems.^4,5,6,7,8,9,10,11,12^ Findings in studies from 2010 to 2017^13,14,15^ suggested that the increased risk of mental health impairment in this population includes increased risk of psychiatric disorders and increased use of psychotropic drugs. While children born preterm have an increased risk of attention deficit hyperactivity disorder (ADHD) and autism,^5,12,16,17,18,19,20^ there is still uncertainty as to whether they have an increased risk of depression and anxiety^21,22,23,24^ or psychotic and bipolar disorders,^10,11,12,25^ with studies finding divergent results.

The increased risk of mental health impairment among children born preterm is found throughout their lifespan, from childhood through adolescence and into adulthood.^8^ However, to our knowledge, a minority of the studies in this field have targeted adolescence, a period during which many mental health difficulties emerge.^26^

The aim of this study was to assess the association between individuals’ degree of preterm birth and prescriptions of psychotropic drugs during adolescence and into young adulthood. Additionally, we wanted to assess associations within sibling groups to reduce confounding due to factors shared within families^27,28,29,30^ and explore how associations for the different drug types varied by age and sex.

## Methods

This cohort study was assessed and approved by the Regional Committees for Medical and Health Research Ethics (REC) and the Norwegian Data Protection Authority. A waiver of informed consent was granted by REC because of the difficulty of obtaining consent owing to population size. This study followed the Strengthening the Reporting of Observational Studies in Epidemiology (STROBE) reporting guideline for cohort studies.

### Study Design

This study was based on a registry data linkage among the Medical Birth Registry of Norway (MBRN),^31^ Norwegian Prescription Database (NORPD),^32^ and Statistics Norway.^33^ We included all Norwegians who were born from 1989 through 1998, had a gestational age (GA) of from 23 weeks to less than 45 weeks, had a birthweight greater than 400 g, had no registered congenital birth defects, were alive at age 10 years, and had registered maternal variables. We excluded individuals for whom the size of the discrepancy between birth weight and gestational age suggested that it was most likely associated with errors in registrations. The MBRN includes virtually all births in the country and provides information on maternal and neonatal health, education, and demographic characteristics at birth. The information was linked using a unique personal identification number, and siblings were identified using the unique identification number for each mother, provided by Statistics Norway. Information from the NORPD, established in 2004, provided a continuously updated listing of all prescribed drugs dispensed by pharmacies. Data from Statistics Norway provided dates of emigration and death.

### Follow-up

All study participants were followed up with annual assessments from 2004 through 2016. Participants were followed up from age 10 years until the year of emigration, death, or 24th birthday, whichever occurred first.

### Exposure

We measured GA by completed weeks of gestation at birth, as recorded in the MBRN according to the mother’s last menstrual period. We categorized GA into 4 groups: extremely preterm (GA, 23 weeks and 0 days to 27 weeks and 6 days), very preterm (GA, 28 weeks and 0 days to 31 weeks and 6 days), moderately or late preterm (GA, 32 weeks and 0 days to 36 weeks and 6 days), and full term (GA, 37 weeks and 0 days to 44 weeks and 6 days).

### Outcome

Outcomes were receiving psychotropic drug prescriptions during follow-up period, per data collected from the NORPD. We defined 5 categories of psychotropic drugs, classified according to the Anatomical Therapeutic Chemical (ATC) system (eTable 1 in the Supplement): N06A, antidepressants; N06B, psychostimulants; N05CD, N05CF, and N05CH, hypnotics and sedatives; N05B, anxiolytics; and N05A, antipsychotics.^34^ In addition to recording specific prescriptions for each drug type for individuals, we recorded if individuals were prescribed any of the 5 types. Prescription status (ie, at least 1 prescription vs no prescription) was recorded each year during follow-up according to age for each of the 5 types of psychotropic drugs and for any prescription. Based on these annual registrations, we also constructed measures of prescription vs no prescription for each of the drug groups for the entire follow-up period (ie, ages 10-23 years), early adolescence (ie, ages 10-16 years), and late adolescence or early adulthood (ie, ages 17-23 years).

We constructed an alternative outcome for antipsychotics, which most likely were prescribed for psychotic disorders, by recording high-dose prescriptions for the drugs aripiprazole, olanzapine, and quetiapine. High dose was defined as more than 180 defined daily doses (DDD) per year; DDD is an international standardized unity for drug consumption and is defined as the assumed mean maintenance dose per day for a drug used for its main indication among adults.

### Covariates

We included covariates considered as potential confounders in the association between gestational age and mental health. Child variables collected from the NMBR included sex, birth year, birth weight, and multiple birth status. We created a *z* score for birth weight using Marsal et al’s fetal growth standards^35^ and identified individuals with birth weights more than 6 SDs less than mean z score or more than 3 SDs greater than mean *z* score by gestational age. Maternal variables, including parity and relationship status, were also collected from NMBR. Maternal age, country of birth, and highest education attained at the time of the child’s birth were obtained from Statistics Norway. Details of covariates are presented in eTable 2 in the Supplement. Analyses were performed both with and without covariates.

### Statistical Analysis

In the population sample, we used logistic regression models to compare psychotropic drug prescription from age 10 to 23 years in each of the preterm groups with prescription in the term group during the follow-up period. All analyses were repeated for each of the 5 drug types and for any drug in unadjusted models and models adjusted for participant’s sex, year of birth, multiple birth status, and birthweight z score and mothers’ parity, relationship status, age in years and age in years squared, educational level, and country of birth. Analyses were repeated for male and female participants separately and using alternative outcomes of early prescription (ie, age 10 to 16 years) and late prescription (ie, age 17 to 23 years). Further adjustment for confounding by parental or family factors was done by comparing drug prescription vs no prescription within maternal sibling groups using conditional logistic regression analyses. All maternal siblings were eligible for the sibling comparison analysis, but only sibling groups differing in both exposure and outcome status were used in the calculation of estimates.

To study the development from ages 10 to 23 years, repeated measurements of psychotropic drug prescription at each age (in years) were compared between each of the preterm groups and the term group using logistic regression with general estimating equation models. All models were stratified by sex and adjusted for participant’s year of birth and multiple birth status and mothers’ parity, relationship status, age in years and age in years squared, educational level, and country of birth. Estimates from these models were used to calculate annual percentages with drug prescription for each gestational age group at 3-year age intervals from age 10 to 23 years.

We performed sensitivity analyses to explore whether exclusion of individuals who died before age 10 years or exclusion of birth defects were associated with changes in our outcome estimates.

We performed all analyses using STATA statistical software version 15.1 (StataCorp), and precision was evaluated with 95% CIs, where estimates within this interval could be described as compatible with data given the model assumptions. Analyses of data were performed from August 2018 through February 2020.

## Results

**Figure 1.**
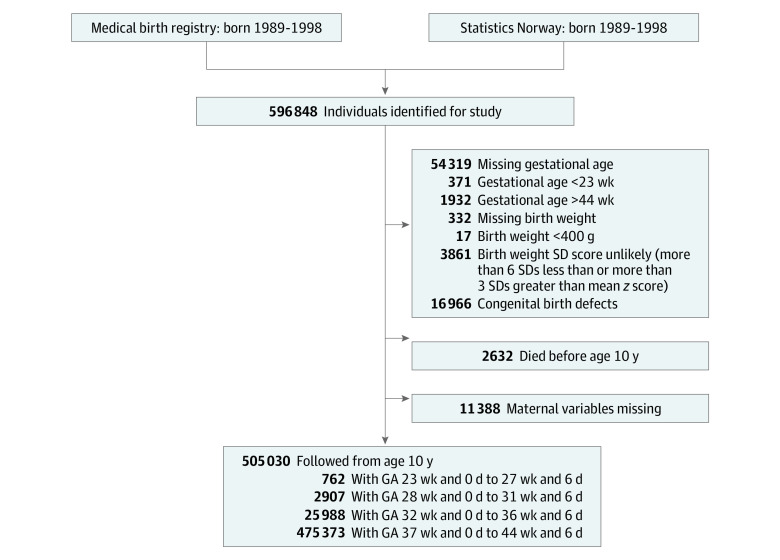
Study Population GA indicates gestational age.

Among 505 030 individuals included in the analyses (259 545 [51.4%] male participants; mean [SD] birth weight, 3533 [580] g) (Figure 1), 762 individuals (0.2%) were extremely preterm, 2907 individuals (0.6%) were very preterm, 25 988 individuals (5.1%) were moderately or late preterm, and 475 373 individuals (94.1%) were full term. Table 1 shows sociodemographic characteristics and perinatal variables for the study population according to GA group. In the study group, 291 754 individuals (57.8%) had at least 1 maternal sibling in the cohort, clustered within 133 623 sibling groups with same mother.

**Table 1.** Sociodemographic Characteristics and Perinatal Variables of Participants

	No. (%)				
	Extremely preterm^a^	Very preterm^b^	Moderately or late preterm^c^	Full term^d^	All groups
Total	762 (0.2)	2 907 (0.6)	25 988 (5.2)	475 373 (94.1)	505 030 (100)
Sex					
Males	381 (50.0)	1621 (55.8)	14 324 (55.1)	243 219 (51.2)	259 545 (51.4)
Females	381 (50.0)	1286 (44.2)	11 664 (44.9)	232 154 (48.8)	245 485 (48.6)
Birthweight, mean (SD), g	901 (202)	1436 (371)	2619 (605)	3600 (498)	3533 (580)
Small for gestational age^e^	21 (2.8)	235 (8.1)	1597 (6.2)	11 288 (2.4)	13 141 (2.6)
Large for gestational age^f^	26 (3.4)	84 (2.9)	1234 (4.8)	12 453 (2.6)	13 797 (2.7)
Mother's relationship status					
Married or cohabitant	682 (89.5)	2565 (88.2)	23 345 (89.8)	436 425 (91.8)	463 017 (91.7)
Other	80 (10.5)	342 (11.8)	2 643 (10.2)	38 948 (8.2)	42 013 (8.3)
Multiple births					
Singletons	583 (76.5)	2190 (75.3)	20 897 (80.4)	467 308 (98.3)	490 978 (97.2)
Twins	150 (19.7)	607 (20.9)	4735 (18.2)	8055 (1.7)	13 547 (2.7)
Triplets or quadruplets	29 (3.8)	110 (3.8)	356 (1.4)	10 (0)	505 (0.1)
Parity					
Primiparae	357 (46.9)	1543 (53.1)	12 765 (49.1)	194 932 (41.0)	209 597 (41.5)
Para					
1	212 (27.8)	756 (26)	7684 (29.6)	171 450 (36.1)	180 102 (35.7)
2	130 (17.1)	417 (14.3)	3779 (14.5)	81 073 (17.1)	85 399 (16.9)
3	43 (5.6)	145 (5.0)	1229 (4.7)	20 594 (4.3)	22 011 (4.4)
≥4	20 (2.6)	46 (1.6)	531 (2.1)	7 324 (1.5)	7 921 (1.6)
Maternal age, mean (SD), y	29.6 (5.3)	29.2 (5.4)	28.8 (5.3)	28.7 (5.0)	28.7 (5.0)
Maternal education					
Primary education	257 (33.7)	960 (33.0)	8504 (32.7)	134 113 (28.2)	143 834 (28.5)
Secondary education	295 (38.7)	1190 (40.9)	10 795 (41.5)	203 424 (42.8)	215 704 (42.7)
Higher education	210 (27.6)	757 (26.0)	6689 (25.7)	137 836 (29.0)	145 492 (28.8)
Maternal country of birth					
Norway	685 (89.9)	2627 (90.4)	23 728 (91.3)	439 084 (92.4)	466 124 (92.3)
Other	77 (10.1)	280 (9.6)	2260 (8.7)	36 289 (7.6)	38 906 (7.7)
Psychotropic drug^g^					
Any	239 (31.4)	697 (24.0)	5386 (20.7)	92 462 (19.5)	98 784 (19.6)
Antidepressant	102 (13.4)	286 (9.8)	2359 (9.1)	41 770 (8.8)	44 517 (8.8)
Psychostimulant	82 (10.8)	227 (7.8)	1420 (5.5)	21 467 (4.5)	23 196 (4.6)
Anxiolytic	87 (11.4)	216 (7.4)	1587 (6.1)	26 465 (5.6)	28 355 (5.6)
Hypnotic or sedative	105 (13.8)	313 (10.8)	2546 (9.8)	42 723 (9.0)	45 687 (9.1)
Antipsychotic	59 (7.7)	138 (4.8)	1014 (3.9)	16 821 (3.5)	18 032 (3.6)
Sibling in cohort^h^	398 (52.2)	1632 (65.1)	15 510 (59.7)	274 214 (57.7)	291 754 (57.8)

^a^ Gestational age, 23 wk and 0 d to 27 wk and 6 d.

^b^ Gestational age, 28 wk and 0 d to 31 wk and 6 d.

^c^ Gestational age, 32 wk and 0 d to 36 wk and 6 d.

^d^ Gestational age, 37 wk and 0 d to 44 wk and 6 d.

^e^ Birth weight <2.5th percentile for gestational age.

^f^ Birth weight >97.5th percentile for gestational age.

^g^ For entire period (ie, ages 10-23 years).

^h^ Individuals with 1 or more maternal siblings in the study group.

Compared with individuals born at full term, individuals born preterm had significantly higher odds of prescription for all the psychotropic drug groups at ages 10 to 23 years (Table 2), increasing from the moderately preterm groups to the extremely preterm groups. The unadjusted estimates (Table 2) did not change substantially after adjusting for potential confounding factors (Table 2). The extremely preterm group had higher odds of being prescribed any kind of psychotropic drug compared with the term group (adjusted odds ratio [OR], 2.1; 95% CI, 1.8-2.4). Estimates for the different drug types were similar, with a higher adjusted OR for the extremely preterm group (antidepressants: OR, 1.7; 95% CI, 1.4-2.1; psychostimulants: OR, 2.7; 95% CI, 2.1-3.4; hypnotics or sedatives: OR, 1.7; 95% CI, 1.4-2.1; anxiolytics: OR, 2.4; 95% CI, 1.9-3.0; and antipsychotics: OR, 2.4; 95% CI, 1.9-3.2). Estimates were smaller for other preterm groups, including the moderately or late preterm group (any drug: OR, 1.1; 95% CI, 1.1-1.2; antidepressants: OR, 1.1; 95% CI, 1.0-1.1; psychostimulants: OR, 1.2; 95% CI, 1.1-1.2; hypnotics or sedatives: OR, 1.1; 95% CI, 1.1-1.2; anxiolytics: OR, 1.1; 95% CI, 1.1-1.2; antipsychotics: OR, 1.1; 95% CI, 1.1-1.2). Overall findings were similar for male and female participants (eTable 3 in the Supplement). Estimates from Table 2 are shown in eTable 4 in the Supplement; eTable 3 in the Supplement shows risk differences (marginal effects, with covariates as observed).

**Table 2.** Association Between Gestational Age and Prescription of Psychotropic Drugs

Gestational age	OR (95% CI)		
	Unadjusted	Adjusted^a^	Sibling comparison
Any drug			
Extremely preterm^b^	2.0 (1.7-2.3)	2.1 (1.8-2.4)	1.8 (1.2-2.8)
Very preterm^c^	1.4 (1.2-1.5)	1.4 (1.3-1.5)	1.2 (0.9-1.5)
Moderate/late preterm^d^	1.1 (1.1-1.1)	1.1 (1.1-1.2)	1.0 (0.9-1.1)
Full term^e^	1.0 (1.0-1.0)	1.0 (1.0-1.0)	1.0 (1.0-1.0)
Antidepressant			
Extremely preterm^b^	1.7 (1.4-2.1)	1.7 (1.4-2.1)	1.2 (0.6-2.2)
Very preterm^c^	1.2 (1.1-1.4)	1.2 (1.1-1.4)	1.1 (0.8-1.5)
Moderately or late preterm^d^	1.1 (1.0-1.1)	1.1 (1.0-1.1)	1.0 (0.9-1.1)
Full term^e^	1.0 (1.0-1.0)	1.0 (1.0-1.0)	1.0 (1.0-1.0)
Psychostimulant			
Extremely preterm^b^	2.5 (2.0-3.2)	2.7 (2.1-3.4)	4.0 (1.7-9.1)
Very preterm^c^	1.7 (1.5-2.0)	1.7 (1.5-2.0)	1.9 (1.3-2.9)
Moderately or late preterm^d^	1.2 (1.1-1.3)	1.2 (1.1-1.2)	1.1 (1.0-1.3)
Full term^e^	1.0 (1.0-1.0)	1.0 (1.0-1.0)	1.0 (1.0-1.0)
Hypnotic or sedative			
Extremely preterm^b^	1.6 (1.3-2.0)	1.7 (1.4-2.1)	1.5 (0.9-2.6)
Very preterm^c^	1.3 (1.1-1.4)	1.3 (1.1-1.4)	1.0 (0.7-1.3)
Moderately or late preterm^d^	1.1 (1.1-1.2)	1.1 (1.1-1.2)	1.0 (0.9-1.2)
Full term^e^	1.0 (1.0-1.0)	1.0 (1.0-1.0)	1.0 (1.0-1.0)
Anxiolytic			
Extremely preterm^b^	2.3 (1.8-2.9)	2.4 (1.9-3.0)	2.2 (1.1-4.1)
Very preterm^c^	1.4 (1.2-1.7)	1.5 (1.3-1.7)	1.1 (0.8-1.6)
Moderately or late preterm^d^	1.1 (1.1-1.2)	1.1 (1.1-1.2)	1.0 (0.9-1.1)
Full term^e^	1.0 (1.0-1.0)	1.0 (1.0-1.0)	1.0 (1.0-1.0)
Antipsychotic			
Extremely preterm^b^	2.4 (1.8-3.1)	2.4 (1.9-3.2)	1.8 (0.8-4.1)
Very preterm^c^	1.4 (1.2-1.7)	1.4 (1.2-1.7)	1.3 (0.9-1.9)
Moderately or late preterm^d^	1.1 (1.1-1.2)	1.1 (1.1-1.2)	1.1 (0.9-1.3)
Term^e^	1.0 (1.0-1.0)	1.9 (1.0-1.0)	1.0 (1.0-1.0)

^a^ Adjusted for participant’s year of birth, multiple birth status, and birthweight *z* score and mothers’ parity, relationship status, age in years and age in years squared, educational level, and country of birth.

^b^ Gestational age, 23 wk and 0 d to 27 wk and 6 d.

^c^ Gestational age, 28 wk and 0 d to 31 wk and 6 d.

^d^ Gestational age, 32 wk and 0 d to 36 wk and 6 d.

^e^ Gestational age, 37 wk and 0 d to 44 wk and 6 d.

Table 2 also shows the results from the within-sibling analyses. Odds of drug prescription by gestational age group were lower for most drugs in the sibling analysis compared with the analysis in the entire study population and were not significant for several drugs in very and moderately or late preterm groups. For example, the OR for any prescription in the sibling analysis was 1.8 (95% CI, 1.2-2.8) in the very preterm group and 1.0 (95% CI, 0.9-1.1) in the moderately or late preterm group. In contrast, there were associations between all degrees of preterm birth and prescription of psychostimulants in the sibling analysis. The OR for psychostimulants in the sibling analysis was 4.0 (95% CI, 1.7-9.1) in the extremely preterm group, 1.9 (95% CI, 1.3-2.9) in the very preterm group, and 1.1 (95% CI, 1.0-1.3) in the moderately or late preterm group.

**Figure 2.**
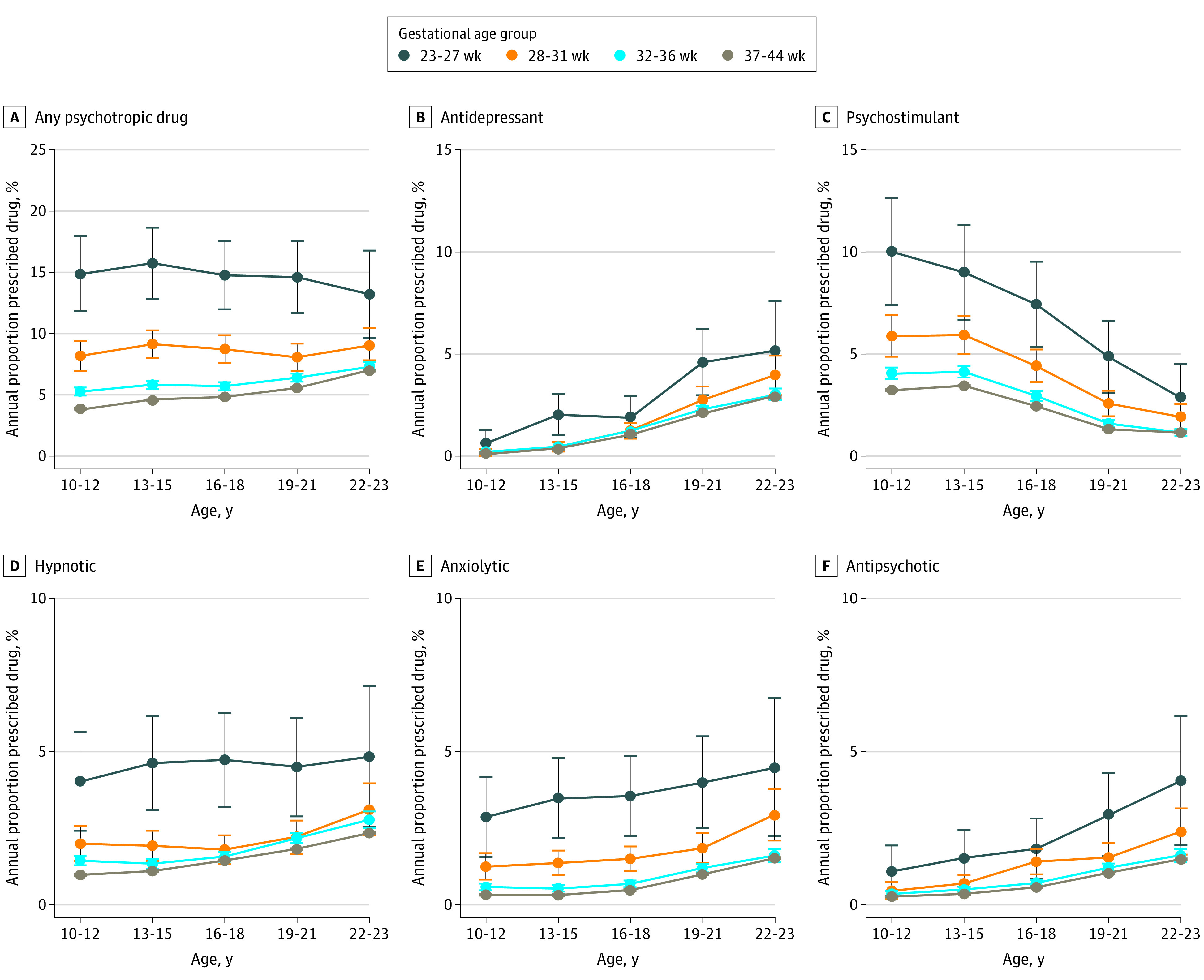
Percentage of Male Participants With at Least 1 Prescription by Gestational Age

**Figure 3.**
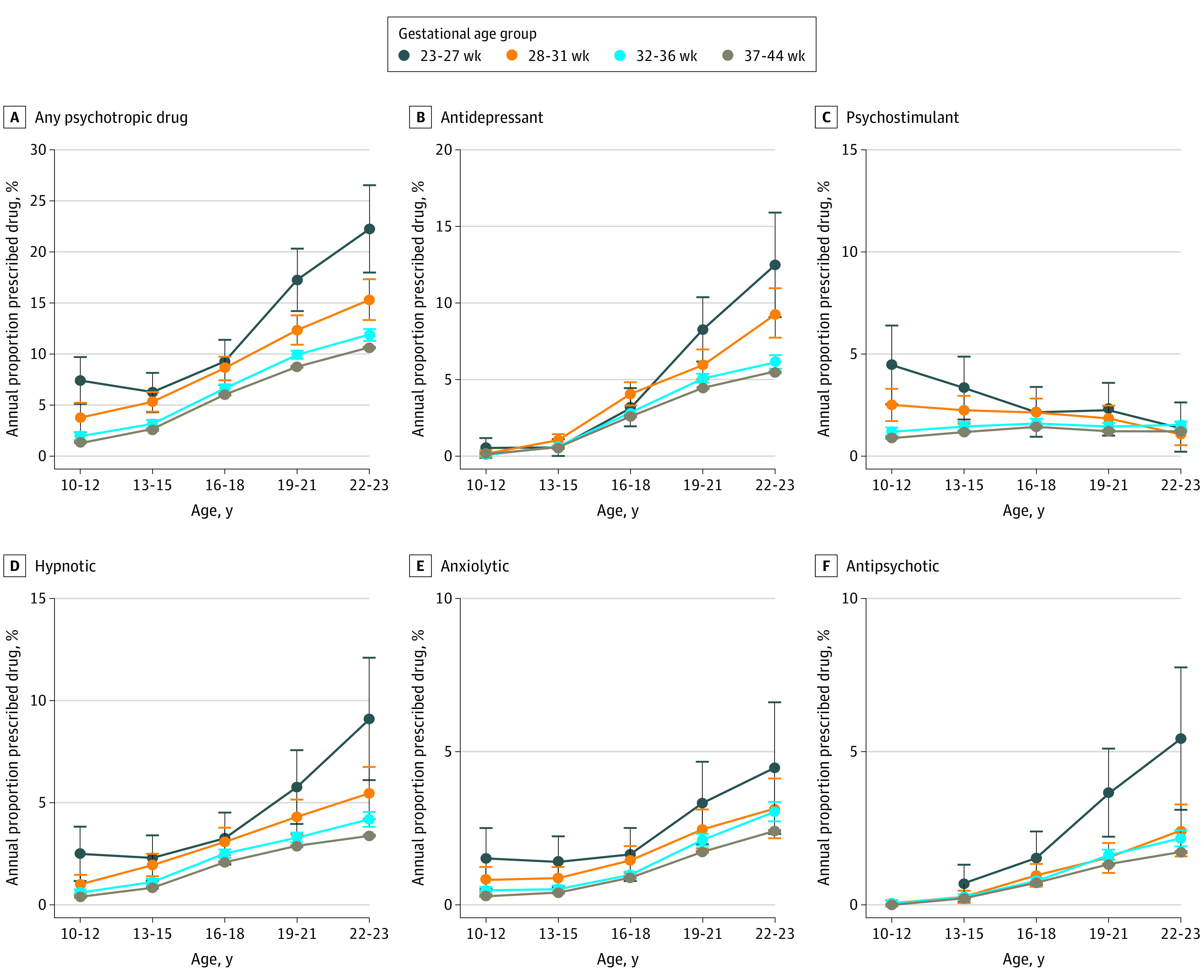
Percentage of Female Participants With at Least 1 Prescription by Gestational Age

Figure 2 and Figure 3 illustrate a dose-response association between gestational age and drug prescription throughout the follow-up period as well as substantial differences in prescription patterns between male and female participants by age. Prescription of psychotropic drugs increased with age for the preterm and term groups; however, prescription of psychostimulants decreased with increasing age. At ages 10 to 12 years and 13 to 15 years, more boys than girls in all gestational age groups were prescribed any psychotropic medication, with the greatest differences in psychostimulants (for example, the annual percentage prescribed in the extremely preterm group was 10.1% (95% CI, 7.4% to 12.6%) in boys and 4.5% (95% CI, 2.5% to 6.4%) in girls at ages 10 to 12 years). In contrast, at ages 19 to 21 years and 22 to 23 years, the situation was reversed, with a greater increase in psychotropic prescriptions over time for female participants than for male participants in all gestational age groups, with the greatest increase in antidepressants. For example, the estimated percentage with annual prescriptions of antidepressants in the extremely preterm group increased by 12.6 percentage points (95% CI, 9.1 percentage points to 16.1 percentage points) in females and 4.4 percentage points (95% CI, 2.1 percentage points to 6.8 percentage points) in males from ages 10 to 12 years to ages 22 to 23 years. Corresponding increases in the full-term group were 5.7 percentage points (95% CI, 5.6 percentage points to 5.8 percentage points) for females and 2.8 percentage points (95% CI, 2.7 percentage points to 2.9 percentage points) for males.

### Additional Analyses

Results for drug prescription during age periods 10 to 16 years and 17 to 23 years for male and female participants separately (eTable 5 in the Supplement) are similar to the main results (Table 2). However, ORs were higher for prescriptions of most drugs for preterm boys during age period 10 to 16 years.

To assess whether the observed use of antipsychotics was associated with psychosis, we performed analyses on the alternative outcome of antipsychotics. Although only 2018 individuals (0.4% of the population sample and 11% of individuals prescribed antipsychotic drugs) received aripiprazole, olanzapine, or quetiapine at high doses (ie, >180 DDD for at least 1 year), the odds of such prescription were increased for the extremely preterm group (OR, 3.5; 95% CI, 1.9-6.6) compared with the full-term group. There were too few individuals for sibling comparison.

Sensitivity analyses found that 1313 children (37.9%) among 3467 children in the original population sample (before exclusion) who died before age 10 years were born preterm; 1210 of them (92.2%) died before age 1 year (eTable 6 in the Supplement). In analyses of the group including individuals with congenital birth defects, there was no change the association between GA and prescription of any of the studied drugs (eTable 7 in the Supplement).

## Discussion

This cohort study found higher rates of prescription of psychotropic drugs among adolescents and young adults born preterm compared with their peers born at full term, an indicator associated with mental health impairment in this group. Adolescents and young adults born preterm had higher odds, compared with those born at full term, for prescription of all psychotropic drugs studied: antidepressants, psychostimulants, anxiolytics, hypnotics and sedatives, and antipsychotics. Odds were greatest for extremely and very preterm birth groups but was also found for moderately or late preterm groups. There was no change in this association after adjustment for perinatal and parental factors. In analyses comparing siblings, odds were high for extremely preterm birth groups, while lower or not significant for very preterm birth groups and moderately or late preterm birth groups. These findings suggest that confounding factors may play a significant role in the associations under study in these groups. There were some differences in the size of the estimates among male and female participants at different ages, with greater relative odds increases for drug prescription among younger male participants and the largest absolute differences between gestational age groups for prescription of antidepressants among older female participants.

### Findings Compared With Existing Knowledge

Our findings support much of the existing knowledge about preterm birth and the risk of social problems and mental health issues in adolescence and young adulthood. We found associations between preterm birth and prescription of psychotropic drugs, reconcilable with findings from cohort studies from 2016^5^ and 2017^41^ and population-based registry studies from 2009 to 2013.^11,12,13,14^

It has been debated to what extent preterm births are associated with an increased risk of psychotic disorders, an association that research from 2008 to 2013 may suggest.^1,10,11,12,13,14^ D’onofrio et al^12^ found that family conditions were associated with much of this increase in risk but not all. We found, as did a Swedish study from 2010^14^ looking at prescriptions, a dose-response association between degree of preterm birth and prescription of antipsychotic drugs for all ages. However, the ATC group N05A contains several antipsychotic drug types, which are prescribed on various indications. The finding in the present study of a 3-fold increase in odds for prescriptions of aripiprazole, olanzapine, and quetiapine given at high doses in the extremely preterm group may suggest increased risk of psychotic disease in this group. However, this finding must be interpreted with caution given that only 0.4% of the study population received this kind of antipsychotic prescription.

For the group born extremely preterm, estimates from the sibling comparison suggested that the risk of mental health impairment during adolescence and young adulthood may be associated with their premature births. Mental health impairment among individuals born preterm is usually explained by complex interplay among prenatal and perinatal factors, including disturbed brain development and stress responses. The process of injury, often referred to as encephalopathy of prematurity, is associated with inflammation, hypoxic-ischemic lesions, and dysmaturation in the preterm brain, and related to white matter disease and changes in gray matter in the cortex and the deep nuclei.^42^ In addition, being born preterm is often associated with more health care contacts throughout childhood, owing to standardized follow-up programs initiated by the health care system^43^ and increased morbidity. This could be associated with increased awareness and detection of potential adverse outcomes associated with preterm birth, including increased diagnosis of mental disorders and prescription of psychotropic drugs for the most preterm individuals.

The association between GA and prescription in our study was found not only for extremely and very preterm birth, but also for moderately or late preterm groups. For these preterm birth groups (GA, 32 weeks-36 weeks) recent studies have found an increased psychiatric morbidity in adolescence and adulthood, including hospital admissions for psychiatric disorders and psychiatric diagnoses.^11,44^

Similar to the results from other studies, our findings suggest that there may be different mechanisms associated with different types of conditions. D’onofrio et al^12^ found associations between GA and ADHD and autism, but after adjusting with sibling analyses, the increases in risks were smaller for bipolar and psychotic disorder and there was no significant increase in risks for suicide or receipt of social welfare benefits. Lindström et al^18^ also found an unchanged association between GA and use of drugs for ADHD when comparing siblings. This is consistent with our findings, which suggest that many of the mental and social conditions found in the preterm population, with the exception of ADHD, could be associated with confounding factors in the environment or genetics, especially for the later preterm groups. There is evidence suggesting that children born preterm from households with low socioeconomic status are at greater risk for mental health impairment.^11^ This association has been a subject of debate, and a possible explanation for our findings might be that confounding factors play an increasing role with increasing GA. The findings in prescription of psychotropic drugs from ages 10 to 23 years in this study add detail and nuance to existing knowledge. We have not been able to find other similar studies of how the increased use of psychotropic medication for children born preterm varies by drug type, sex, and age. Of particular interest was the prominent increase in prescriptions from ages 16 to 18 years and 19 to 21 years found among female participants born extremely preterm .

### Strengths

Strengths of our study include the study design, with a large, naturally selected population distributed across all gestational ages, with the possibility to follow up all participants using high-quality registry data, along with the opportunity to study the population over several years and across participants’ youth. Using sibling design made it possible to adjust for confounding factors shared by siblings.

### Limitations

This cohort study has several limitations. In our registry data, term date was determined based on the mothers last menstrual period, which is a less exact method than using ultrasound measurement.^36,37,38^ The method based on menstrual period has been shown to be associated with overestimations of the true gestational age by 2 or 3 days. However, we consider such misclassification as nondifferential, which would most likely bias the results from the population analyses toward the null.

Prescriptions of a psychotropic drug provides little information about specific psychiatric diagnoses. However, we consider it a sensitive measure of mental health difficulties.^39^

Even if sibling comparison may be an effective way of accounting for unmeasured confounding associated with family background, it has been suggested that this approach may increase selection bias and measurement error.^40^ Random measurement error in the assessment of gestational age may thus be increased when comparing siblings and may lead to an attenuation of estimates in the sibling comparison. However, we consider error in the measurement of gestational age a minor issue owing to standardized procedures and registration.

We had no knowledge of diagnoses other than perinatal conditions, which makes it difficult to rule out confounding from other comorbid conditions present at birth but diagnosed later.

Additionally, these findings may primarily be valid only for survivors, because only those alive at age 10 years were followed. Individuals born preterm were overrepresented among those who died, and we expect that if survival were greater among these individuals, the prescription of psychotropic medication found in our study would also have increased.

## Conclusions

This cohort study found that the prescription of psychotropic drugs was higher among adolescents and young adults who were born preterm compared with those born at term. Children born preterm have increased mental and social risks in adolescent and young adult years. This increased risk may be largely associated with factors involving genetics and childhood environment for the later preterm groups. Trajectories established during adolescence are associated with later mental health status and lifetime opportunities, and this period could thus be an important target for health-promoting measures and preventive measures for this group.

## Notes

Supplement. **eTable 1.** Overview of Registered Active Substances in the Anatomical Therapeutic Chemical System in Norway, September 2020 **eTable 2.** Overview of Variables **eTable 3.** Odds Ratios with 95% CIs for Prescription of Psychotropic Drugs According to Gestational Age, Stratified by Sex **eTable 4.** Risk Differences With 95% CIs for Prescription, Total Population and Stratified by Sex **eTable 5.** Odds Ratios With 95% CIs for Prescription of Psychotropic Drugs According to Gestational Age, Stratified by Age During Follow-Up and Sex **eTable 6.** Demographic Characteristics for Individuals Who Died Before Age 10 Years **eTable 7.** Sensitivity Analysis Including Individuals With Congenital Birth Defects
