# Supplementary material for: Association of Preterm Birth With Prescription of Psychotropic Drugs in Adolescence and Young Adulthood
Source: JAMA Netw Open. 2021 Mar 12;4(3):e211420. doi: 10.1001/jamanetworkopen.2021.1420 (PMC7955275; doi:10.1001/jamanetworkopen.2021.1420)
Supplement: Supplement. — eTable 1. Overview of Registered Active Substances in the Anatomical Therapeutic Chemical System in Norway, September 2020 eTable 2. Overview of Variables eTable 3. Odds Ratios with 95% CIs for Prescription of Psychotropic Drugs According to Gestational Age, Stratified by Sex eTable 4. Risk Differences With 95% CIs for Prescription, Total Population and Stratified by Sex eTable 5. Odds Ratios With 95% CIs for Prescription of Psychotropic Drugs According to Gestational Age, Stratified by Age During Follow-Up and Sex eTable 6. Demographic Characteristics for Individuals Who Died Before Age 10 Years eTable 7. Sensitivity Analysis Including Individuals With Congenital Birth Defects [file jamanetwopen-e211420-s001.pdf]

Supplemental Online Content

Bachmann CS, Risnes K, Bjørngaard JH, Schei J, Pape K. Association of preterm birth with prescription of psychotropic drugs in adolescence and young adulthood. *JAMA Netw Open*. 2021;4(3):e211420. doi:10.1001/jamanetworkopen.2021.1420

eTable 1. Overview of Registered Active Substances in the Anatomical Therapeutic Chemical System in Norway, September 2020

eTable 2. Overview of Variables

eTable 3. Odds Ratios with 95% CIs for Prescription of Psychotropic Drugs According to Gestational Age, Stratified by Sex

eTable 4. Risk Differences With 95% CIs for Prescription, Total Population and Stratified by Sex

eTable 5. Odds Ratios With 95% CIs for Prescription of Psychotropic Drugs According to Gestational Age, Stratified by Age During Follow-Up and Sex

eTable 6. Demographic Characteristics for Individuals Who Died Before Age 10 Years

eTable 7. Sensitivity Analysis Including Individuals With Congenital Birth Defects

This supplemental material has been provided by the authors to give readers additional information about their work.

© 2021 Bachmann CS et al. *JAMA Network Open.*

eTable 1. Overview of Registered Active Substances in the Anatomical Therapeutic Chemical System in Norway, September 2020

N06A Antidepressants:	Non-selective monoamine reuptake inhibitors: clomipramine, trimipramine, amitriptyline, nortriptyline, doxepin<br>Selective serotonin reuptake inhibitors: fluoxetine, citalopram, paroxetine, sertraline, fluvoxamine, escitalopram<br>Monoamine oxidase A inhibitors: moclobemide<br>Other antidepressants: mianserin, mirtazapine, bupropion, venlafaxine, reboxetine, duloxetine, vortioxetine, esketamine
N06B Psychostimulants:	Centrally acting sympathomimetics: amphetamine, dexamphetamine, methylphenidate, modafinil, atomoxetine, lisdexamphetamine<br>Xanthine derivatives: caffeine<br>Other psychostimulants: piracetam, idebenone
N05B Anxiolytics:	Benzodiazepine derivatives: diazepam, oxazepam, alprazolam<br>Diphenylmethane derivatives: hydroxyzine<br>Azaspirodecandion derivatives: buspirone
N05C Hypnotics and sedatives:	Benzodiazepine derivatives: nitrazepam, midazolam<br>Benzodiazepine-like agents: zopiclone, zolpidem<br>Melatonin receptor agonists: melatonin<br>Other hypnotics and sedatives: clomethiazole, scopolamine, valarianae radix, dexmedetomidine
N05A Antipsychotics:	Phenothiazines with aliphatic side-chain: levomepromazine<br>Phenothiazines with piperazine structure: perphenazine, prochlorperazine<br>Butyrophenone derivatives: haloperidol, droperidol<br>Indole derivatives: sertindole, ziprasidone, lurasidone<br>Thioxanthene derivatives: flupentixol, chlorprothixene, zuchlopenthixol<br>Diazepines, oxazepines, thiazepines and oxepines: clozapine, olanzapine, quetiapine<br>Benzamides: amisulpride<br>Lithium: lithium<br>Other antipsychotics: risperidone, aripiprazole, paliperidone, cariprazine, brexpiprazole

© 2021 Bachmann CS et al. *JAMA Network Open.*

eTable 2. Overview of Study Variables:

Variables	Registy	Definitions
Child variables at birth:		
Gestational age	Medical Birth Registry	Completed weeks, according to mothers last menstrual period;<br>4 categories: 23+0-27+6, 28+0-31+6, 32+0-36+6 and 37+0-44+6.
Date of birth	Medical Birth Registry	Used to calculate age in years
Birthweight	Medical Birth Registry	Used to calculate Z-score
Sex	Medical Birth Registry	Boys and girls
Multiple births	Medical Birth Registry	2 categories: Multiples or not
Parental variables:		At the time of the child’s birth
Mother's parity	Medical Birth Registry	Mother's parity at time of birth,<br>5 categories: para 0, 1, 2, 3 and 4+.
Mother's relationship status	Medical Birth Registry	At time of birth:<br>2 categories: married/ cohabitant or other (single, unmarried, divorced, widowed)
Mother's age	Medical Birth Registry	Age at time of birth:<br>Age in years<br>Age in years squared
Mother's education	Statistics Norway	Highest attained education at birth:<br>3 categories: primary education only, secondary education (high school) or higher education
Mother's country of birth	Statistics Norway	2 categories: Norway or any other country
Child variables during follow-up:		
Death	Statistics Norway	Follow-up censored at month of death
Prescriptions of psychotropic drugs	Norwegian Prescription Database	Prescription of ATC-groups:<br>N06A antidepressants,<br>N06B psychostimulants,<br>N05B anxiolytics,<br>N05CD/ N05CF/ N05CH Hypnotics and sedatives<br>N05A antipsychotics

© 2021 Bachmann CS et al. *JAMA Network Open.*

eTable 3. Odds Ratios with 95% CIs for Prescription of Psychotropic Drugs According to Gestational Age, Stratified by Sex

Estimates from model adjusted for year of birth, multiple births, parity, mothers' relationship status, mother's age in years and age in years squared, mother's educational level, country of birth and child's birthweight z-score.

	aOR Boys	aOR Girls	P (interaction)[a]
**Any:**			0,12
Extremely preterm	2.3 (1.8 to 2.9)	1.8 (1.5 to 2.3)	
Very preterm	1.4 (1.3 to 1.6)	1.3 (1.1 to 1.5)	
Moderate/ late preterm	1.1 (1.1 to 1.2)	1.1 (1.0 to 1.2)	
Term	1.0 (1.0 to 1.0)	1.0 (1.0 to 1.0)	
**Antidepressants:**			0,54
Extremely preterm	1.6 (1.1 to 2.3)	1.8 (1.4 to 2.3)	
Very preterm	1.2 (1.0 to 1.4)	1.3 (1.1 to 1.5)	
Moderate/ late preterm	1.1 (1.0 to 1.1)	1.1 (1.0 to 1.2)	
Term	1.0 (1.0 to 1.0)	1.0 (1.0 to 1.0)	
**Psychostimulants:**			0,23
Extremely preterm	2.8 (2.1 to 3.8)	2.4 (1.6 to 3.5)	
Very preterm	1.7 (1.5 to 2.1)	1.6 (1.2 to 2.0)	
Moderate/ late preterm	1.2 (1.1 to 1.3)	1.1 (1.0 to 1.3)	
Term	1.0 (1.0 to 1.0)	1.0 (1.0 to 1.0)	
**Hypnotics/ sedatives:**			0,19
Extremely preterm	1.8 (1.4 to 2.5)	1.6 (1.2 to 2.2)	
Very preterm	1.2 (1.0 to 1.4)	1.4 (1.2 to 1.6)	
Moderate/ late preterm	1.1 (1.0 to 1.2)	1.2 (1.1 to 1.3)	
Term	1.0 (1.0 to 1.0)	1.0 (1.0 to 1.0)	
**Anxiolytics:**			**0,03**
Extremely preterm	3.2 (2.3 to 4.4)	1.8 (1.3 to 2.5)	
Very preterm	1.6 (1.3 to 1.9)	1.3 (1.1 to 1.6)	
Moderate/ late preterm	1.2 (1.1 to 1.3)	1.1 (1.1 to 1.2)	
Term	1.0 (1.0 to 1.0)	1.0 (1.0 to 1.0)	
**Antipsychotics:**			0,8
Extremely preterm	2.2 (1.4 to 3.3)	2.7 (1.9 to 3.8)	
Very preterm	1.4 (1.1 to 1.8)	1.4 (1.1 to 1.7)	
Moderate/ late preterm	1.1 (1.0 to 1.2)	1.2 (1.1 to 1.3)	
Term	1.0 (1.0 to 1.0)	1.0 (1.0 to 1.0)	

[a]p value for statistical interaction between sex and gestational age group,

© 2021 Bachmann CS et al. *JAMA Network Open.*

eTable 4. Risk Differences With 95 % CIs for Prescription, Total Population and Stratified by Sex

Estimates (marginal effects) calculated as average marginal effects from model (same as adjusted model in Table 2 and eTable3) adjusted for year of birth, multiple births, parity, mothers' relationship status, mother's age in years and age in years squared, mother's educational level, country of birth and child's birthweight z-score.

	**RD (%-points)**<br>**All**	**RD (%-points)**<br>**Boys**	**RD (%-points)**<br>**Girls**
**Any:**			
Extremely preterm	13.2 (9.8 to 16.5)	14.3 (9.6 to 18.9)	11.9 (7.1 to 16.7)
Very preterm	4.9 (3.3 to 6.4)	5.6 (3.6 to 7.6)	4.5 (2.0 to 6.9)
Moderate/ late preterm	1.4 (0.9 to 1.9)	1.6 (1.0 to 2.3)	1.6 (0.8 to 2.5)
Term	0.0 (0.0 to 0.0)	0.0 (0.0 to 0.0)	0.0 (0.0 to 0.0)
**Antidepressants:**			
Extremely preterm	5.4 (2.8 to 7.9)	3.4 (0.4 to 6.5)	7.3 (3.3 to 11.3)
Very preterm	1.4 (0.2 to 2.5)	0.9 (-0.4 to 2.1)	2.6 (0.7 to 4.5)
Moderate/ late preterm	0.4 (0.0 to 0.8)	0.3 (-0.1 to 0.7)	1.1 (0.4 to 1.7)
Term	0.0 (0.0 to 0.0)	0.0 (0.0 to 0.0)	0.0 (0.0 to 0.0)
**Psychostimulants:**			
Extremely preterm	6.4 (4.2 to 8.6)	8.5 (5.1 to 11.9)	4.1 (1.4 to 6.8)
Very preterm	3.0 (2.1 to 4.0)	3.7 (2.3 to 5.1)	1.8 (0.6 to 3.0)
Moderate/ late preterm	0.9 (0.6 to 1.1)	1.0 (0.6 to 1.4)	0.4 (0.1 to 0.8)
Term	0.0 (0.0 to 0.0)	0.0 (0.0 to 0.0)	0.0 (0.0 to 0.0)
**Hypnotics/ sedatives:**			
Extremely preterm	5.5 (3.0 to 8.1)	5.5 (2.0 to 8.9)	5.6 (1.8 to 9.4)
Very preterm	2.0 (0.9 to 3.2)	1.1 (-0.3 to 2.5)	3.5 (1.5 to 5.4)
Moderate/ late preterm	1.0 (0.6 to 1.4)	0.6 (0.2 to 1.1)	1.7 (1.0 to 2.3)
Term	0.0 (0.0 to 0.0)	0.0 (0.0 to 0.0)	0.0 (0.0 to 0.0)
**Anxiolytics:**			
Extremely preterm	6.5 (4.2 to 8.9)	8.0 (4.7 to 11.3)	5.0 (1.7 to 8.4)
Very preterm	2.0 (1.1 to 3.0)	2.2 (1.0 to 3.4)	2.1 (0.5 to 3.7)
Moderate/ late preterm	0.6 (0.3 to 0.9)	0.6 (0.2 to 1.0)	0.8 (0.3 to 1.3)
Term	0.0 (0.0 to 0.0)	0.0 (0.0 to 0.0)	0.0 (0.0 to 0.0)
**Antipsychotics:**			
Extremely preterm	4.6 (2.6 to 6.6)	3.4 (0.9 to 5.9)	5.8 (2.7 to 8.9)
Very preterm	1.3 (0.5 to 2.1)	1.3 (0.3 to 2.3)	1.3 (0.1 to 2.5)
Moderate/ late preterm	0.4 (0.1 to 0.6)	0.3 (0.0 to 0.6)	0.6 (0.2 to 1.0)
Term	0.0 (0.0 to 0.0)	0.0 (0.0 to 0.0)	0.0 (0.0 to 0.0)

© 2021 Bachmann CS et al. *JAMA Network Open.*

eTable 5. Odds Ratios With 95% CIs for Prescription of Psychotropic Drugs According to Gestational Age, Stratified by Age During Follow-Up and Sex

	Total[a]	Any:		Antidepressants:		Psychostimulants:		Anxiolytics:		Hypnotics/ sedatives:		Antipsychotics:	
		Number	OR (95% CI)[b]	Number	OR (95% CI)	Number	OR (95% CI)	Number	OR (95% CI)	Number	OR (95% CI)	Number	OR (95% CI)
**Boys 10-16:**													
Extremely preterm [c]	381	87	3.4 (2.6 to 4.3)	13	3.0 (1.7 to 5.2)	52	3.0 (2.2 to 4.0)	28	6.0 (4.1 to 8.9)	30	2.7 (1.9 to 4.0)	9	2.6 (1.3 to 5.1)
Very preterm [d]	1 621	228	1.9 (1.6 to 2.2)	22	1.2 (0.8 to 1.8)	147	1.8 (1.5 to 2.2)	50	2.5 (1.9 to 3.3)	64	1.4 (1.1 to 1.8)	27	1.9 (1.3 to 2.8)
Moderately/ late preterm [e]	14 323	1 346	1.2 (1.1 to 1.3)	166	1.0 (0.9 to 1.2)	868	1.2 (1.1 to 1.3)	259	1.5 (1.3 to 1.7)	443	1.1 (1.0 to 1.2)	144	1.2 (1.0 to 1.4)
Term [f]	243 178	18 852	1.0 (1.0 to 1.0)	2 793	1.0 (1.0 to 1.0)	11 731	1.0 (1.0 to 1.0)	3 002	1.0 (1.0 to 1.0)	6 729	1.0 (1.0 to 1.0)	2 036	1.0 (1.0 to 1.0)
**Boys 17-23:**													
Extremely preterm	379	90	2.2 (1.7 to 2.8)	29	1.5 (1.1 to 2.3)	37	3.2 (2.2 to 4.5)	28	2.7 (1.8 to 3.9)	34	1.7 (1.2 to 2.5)	23	2.5 (1.6 to 3.9)
Very preterm	1 618	278	1.4 (1.2 to 1.6)	100	1.2 (1.0 to 1.4)	101	1.9 (1.5 to 2.3)	75	1.5 (1.2 to 1.9)	102	1.1 (0.9 to 1.4)	63	1.5 (1.2 to 1.9)
Moderately/ late preterm	14 312	2 092	1.1 (1.1 to 1.2)	836	1.1 (1.0 to 1.2)	590	1.2 (1.1 to 1.3)	499	1.1 (1.0 to 1.2)	909	1.1 (1.0 to 1.2)	423	1.1 (1.0 to 1.2)
Term	242 987	32 687	1.0 (1.0 to 1.0)	13 564	1.0 (1.0 to 1.0)	8 134	1.0 (1.0 to 1.0)	7 854	1.0 (1.0 to 1.0)	14 480	1.0 (1.0 to 1.0)	6 557	1.0 (1.0 to 1.0)
**Girls 10-16:**													
Extremely preterm	380	39	1.6 (1.2 to 2.3)	9	1.1 (0.6 to 2.2)	17	2.3 (1.4 to 3.8)	11	1.8 (1.0 to 3.3)	16	1.6 (1.0 to 2.7)	7	2.4 (1.2 to 5.2)
Very preterm	1 286	131	1.6 (1.3 to 1.9)	39	1.4 (1.0 to 1.9)	44	1.7 (1.2 to 2.3)	38	1.8 (1.3 to 2.5)	58	1.7 (1.3 to 2.2)	11	1.1 (0.6 to 2.0)
Moderately/ late preterm	11 662	864	1.1 (1.0 to 1.2)	269	1.1 (0.9 to 1.2)	289	1.2 (1.1 to 1.3)	216	1.1 (1.0 to 1.3)	370	1.2 (1.1 to 1.3)	93	1.0 (0.8 to 1.3)
Term	232 114	15 880	1.0 (1.0 to 1.0)	5 217	1.0 (1.0 to 1.0)	4 886	1.0 (1.0 to 1.0)	3 774	1.0 (1.0 to 1.0)	6 461	1.0 (1.0 to 1.0)	1 805	1.0 (1.0 to 1.0)
**Girls 17-23:**													
Extremely preterm	379	110	1.8 (1.4 to 2.2)	66	1.9 (1.4 to 2.5)	15	1.7 (1.0 to 2.9)	37	1.9 (1.3 to 2.7)	49	1.6 (1.2 to 2.2)	31	2.6 (1.8 to 3.8)
Very preterm	1 284	294	1.2 (1.1 to 1.4)	170	1.3 (1.1 to 1.6)	41	1.3 (1.0 to 1.8)	89	1.3 (1.0 to 1.6)	145	1.4 (1.1 to 1.6)	61	1.4 (1.1 to 1.8)
Moderately/ late preterm	11 652	2 518	1.1 (1.1 to 1.2)	1 378	1.1 (1.1 to 1.2)	326	1.1 (1.0 to 1.3)	768	1.2 (1.1 to 1.2)	1 202	1.2 (1.1 to 1.3)	484	1.2 (1.1 to 1.3)
Term	231 987	46 919	1.0 (1.0 to 1.0)	25 206	1.0 (1.0 to 1.0)	5 870	1.0 (1.0 to 1.0)	13 686	1.0 (1.0 to 1.0)	21 000	1.0 (1.0 to 1.0)	8 281	1.0 (1.0 to 1.0)

[a]Total number of individuals contributing to the group

[b] Odds Ratio with 95 % confidence interval (CI) adjusted for year of birth, multiple births, parity, mothers' relationship status, mother's age in years and age in years squared, mother's educational level, country of birth and child's birthweight z-score.

[c] Gestational age 23+0-27+6

[d] Gestational age 28+0-31+6

[e] Gestational age 32+0-36+6

[f] Gestational age 37+0-44+6

© 2021 Bachmann CS et al. *JAMA Network Open.*

eTable 6. Demographic Characteristics for Individuals Who Died Before Age 10 Years

	Alive at age 10		Dead before age 10	
	N	%	N	%
**Total:**	532,549	99.3	3,467	0.7
**Gestational age groups:**				
Extremely preterm	798	0.2	555	16.0
Very preterm	3,038	0.6	300	8.7
Moderate/ late preterm	27,743	5.2	458	13.2
Term	500,970	94.1	2,154	62.1
**Gender:**				
Boys	273,952	51	1,993	57
Girls	258,597	49	1,474	43
**Mean birthweight, g (SD):**	3529	589	2591	1193
**Small for gestational age[e]:**	14,173	2.7	485	14.0
**Large for gestational age[f]:**	14,486	2.7	77	2.2
**Multiple births:**				
Singeltons	517,874	97.2	3,127	90.2
Twins	14,158	2.7	301	8.7
Triplets/ quadruplets	507	0.1	39	1.1
**Birth defects**	16,131	3.0	835	24.1
**Parity:**				
Primiparae	221,798	41.7	1,342	38.7
Para 1	188,787	35.5	1,143	33.0
Para 2	89,536	16.8	635	18.3
Para 3	23,481	4.4	250	7.2
Para 4 or more	8,947	1.7	97	2.8
**Mother's relationship status:**				
Married/ cohabitant	44,327	8.3	396	11.4
Other	488,222	91.7	3,071	88.6
**Maternal age (mean/SD)**	28.7	5.0	28.5	5.4
**Maternal education:**				
Primary education	148,236	28.5	1,232	36.6
Secondary education	222,327	42.7	1,395	41.5
Higher education	150,254	28.9	738	21.9
**Maternal country of birth:**				
Norway	482,046	90.6	3,108	89.7
Other	49,994	9.4	358	10.3
**Missing variables**	11,732	2.2	102	2.9
**Age at death (median, IQR)**			0.2	0.1, 1.2

© 2021 Bachmann CS et al. *JAMA Network Open.*

eTable 7. Sensitivity Analysis Including Individuals With Congenital Birth Defects

Odds Ratios (OR) with 95 % confidence interval (CI) for the association between gestational age and prescription of psychotropic drugs between ages 10 and 23 years.

	OR[a] (95% CI)	aOR[b] (95% CI)	sOR[c] (95% CI)
**Any:**			
Extremely preterm[d]	1.9 (1.7 to 2.3)	2.0 (1.7 to 2.4)	1.8 (1.2 to 2.7)
Very preterm[e]	1.4 (1.3 to 1.5)	1.4 (1.3 to 1.5)	1.2 (0.9 to 1.4)
Moderate/ late preterm[f]	1.1 (1.1 to 1.1)	1.1 (1.1 to 1.2)	1.0 (0.9 to 1.1)
Term[g]	1.0 (1.0 to 1.0)	1.0 (1.0 to 1.0)	1.0 (1.0 to 1.0)
**Antidepressants:**			
Extremely preterm	1.7 (1.4 to 2.0)	1.7 (1.4 to 2.1)	1.2 (0.6 to 2.1)
Very preterm	1.2 (1.1 to 1.4)	1.2 (1.1 to 1.4)	1.1 (0.8 to 1.4)
Moderate/ late preterm	1.1 (1.0 to 1.1)	1.1 (1.0 to 1.1)	1.0 (0.9 to 1.1)
Term	1.0 (1.0 to 1.0)	1.0 (1.0 to 1.0)	1.0 (1.0 to 1.0)
**Psychostimulants:**			
Extremely preterm	2.5 (2.0 to 3.1)	2.6 (2.0 to 3.3)	4.1 (1.8 to 9.3)
Very preterm	1.8 (1.6 to 2.0)	1.8 (1.5 to 2.0)	1.8 (1.2 to 2.5)
Moderate/ late preterm	1.2 (1.1 to 1.3)	1.2 (1.1 to 1.2)	1.1 (1.0 to 1.3)
Term	1.0 (1.0 to 1.0)	1.0 (1.0 to 1.0)	1.0 (1.0 to 1.0)
**Hypnotics/ sedatives:**			
Extremely preterm	1.7 (1.3 to 2.0)	1.7 (1.4 to 2.1)	1.6 (0.9 to 2.8)
Very preterm	1.3 (1.1 to 1.4)	1.3 (1.1 to 1.5)	0.9 (0.7 to 1.3)
Moderate/ late preterm	1.1 (1.1 to 1.2)	1.1 (1.1 to 1.2)	1.0 (0.9 to 1.1)
Term	1.0 (1.0 to 1.0)	1.0 (1.0 to 1.0)	1.0 (1.0 to 1.0)
**Anxiolytics:**			
Extremely preterm	2.3 (1.8 to 2.8)	2.4 (1.9 to 2.9)	2.0 (1.1 to 3.6)
Very preterm	1.5 (1.3 to 1.7)	1.5 (1.3 to 1.7)	1.2 (0.8 to 1.7)
Moderate/ late preterm	1.1 (1.1 to 1.2)	1.2 (1.1 to 1.2)	1.0 (0.9 to 1.1)
Term	1.0 (1.0 to 1.0)	1.0 (1.0 to 1.0)	1.0 (1.0 to 1.0)
**Antipsychotics:**			
Extremely preterm	2.4 (1.9 to 3.1)	2.5 (1.9 to 3.2)	2.0 (0.9 to 4.5)
Very preterm	1.5 (1.3 to 1.7)	1.5 (1.3 to 1.8)	1.1 (0.8 to 1.6)
Moderate/ late preterm	1.1 (1.1 to 1.2)	1.1 (1.1 to 1.2)	1.1 (1.0 to 1.3)
Term	1.0 (1.0 to 1.0)	1.9 (1.0 to 1.0)	1.0 (1.0 to 1.0)
